# Experiences of identifying pre-school children with disabilities in resource limited settings – an account from Malawi, Pakistan and Uganda

**DOI:** 10.1080/09687599.2023.2181769

**Published:** 2023-02-21

**Authors:** Paul Lynch, Helen M. Nabwera, Harriet M. Babikako, Muneera Rasheed, Kirsten A. Donald, Emmie W. Mbale, Elizabeth Stockdale, Prem Chand, Meta Van den Heuvel, Angelina Kakooza Mwesige, Melissa Gladstone

**Affiliations:** a School of Education & School of Health and Wellbeing, University of Glasgow, Glasgow, UK; b Department of Clinical Sciences, Liverpool School of Tropical Medicine, Liverpool, UK; c Alder Hey Children’s Hospital, Liverpool, UK; d Department of Epidemiology and Biostatistics, Makerere University College of Health Sciences, Kampala, Uganda; e Department of Pediatrics and Child Health, Aga Khan University, Karachi, Pakistan; f Division of Developmental Paediatrics, Department of Paediatrics and Child Health, Red Cross War Memorial Children’s Hospital, University of Cape Town, Cape Town, South Africa; g Neuroscience Institute, University of Cape Town, Cape Town, South Africa; h Department of Pediatrics and Child Health, Kamuzu University of Health Sciences, Blantyre, Malawi; i Hospital for Sick Children, University of Toronto, Toronto, Ontario, Canada; j Department of Paediatrics and Child Health, Makerere University College of Health Sciences, Kampala, Uganda; k Institute of Life Course and Medical Sciences, University of Liverpool, Liverpool, UK

**Keywords:** Malawi, Uganda, Pakistan, neurodevelopmental disabilities, early identification, childhood disability, local beliefs

## Abstract

Although access to effective medical care for acutely sick children has improved globally, the number of children surviving but who may not be thriving due to disability, is increasing. This study aimed to understand the views of health professionals, educators and caregivers of pre-school children with disabilities in Malawi, Pakistan and Uganda regarding early identification, referral and support. Using applied thematic analysis, we identified themes relating to; limited ‘demand’ by caregivers for services; different local beliefs and community perceptions regarding the causes of childhood disability. Themes relating to ‘supply’ of services included inability to respond to community needs, and inadequate training among professionals for identification and referral. Stepwise, approaches provided to the families, community health worker and higher-level services could include training for community and primary care health workers on basic identification techniques and enhanced awareness for families and communities on the importance of early identification of children with disabilities.

## Points of interest

Lack of collaboration between the community and health services may lead to entrenched pessimistic views of what can be done to support children with disabilities - generating a greater mistrust and low parental take-up of vital health services.

If parents do not receive help at the community and clinic level, then, there is a need to move away from trying to provide a ‘specific diagnosis’ to working more on a level of assessing the child’s functioning in terms of what their limitations are and how they can be addressed.

Any identification and referral programme needs to consider the varying local beliefs, the stigma of having a child with a disability and feelings of blame, right from the start.

A stepwise, incremental approaches, ranging from the provision of basic information, such as using brief materials highlighting ‘red flag’ milestones and conditions which are linked to guidance for support to families, community health workers, as well as higher levels of medical services, are likely to work best.

## Introduction

Although access to effective medical care for acutely sick children has improved globally, the number of children surviving but who may not be thriving due to disability, is increasing. This is the case in all settings, but particularly in low- and middle- income countries (LMICs) (United Nations Inter-agency Group for Child Mortality Estimation (UN IGME, 2020). An estimated 53 million children globally (approximately one in ten of all children) have a developmental disorder with the greatest prevalence in LMICs (Global Research on Developmental Disabilities, 2018). For the purposes of this paper, we define developmental disabilities as a group of disorders in which development of the nervous system is disturbed. This includes cerebral palsy, global developmental delay, autism and epilepsy.

Most of the resources put towards improving child health outcomes in these settings have gone towards enhancing acute and emergency care, with programmes such as Helping Babies Breathe (HBB), Integrated Management of Childhood Illness (IMCI) and Emergency Triage Assessment and Treatment (ETAT) being popular training programmes for enabling better identification, routing and care of infants and older children who are acutely unwell (World Health Organization 2005, Kamath-Rayne et al. 2018, Mason, Scherpbier, and Lawe-Davies 2009). As a consequence, there has been a limited focus on the early identification and management of children with neurodevelopmental disorders and disabilities (NDDs) in these settings (Global Research on Developmental Disabilities, 2018). The management of disability in childhood requires a multi-sectoral approach - spanning education, health, social welfare. However, real change requires well-coordinated global action (Olusanya et al. 2022).

Many countries around the world have ratified the UN Convention on the Rights of Persons with Disabilities (CRPD) as well as the UN Convention on the Rights of the Child (CRC). Despite these important policy priorities, in many countries, social, cultural, material, physical or attitudinal barriers to identification and care of children with disabilities still exist, adversely affecting the lives of many families (Global Research on Developmental Disabilities, 2018). It is common for ‘disability’ to be perceived by belief systems that fall into the social, medical or charity models of disability (Stone-MacDonald & Butera, 2012). These local beliefs and practices can adversely affect children with disabilities and their families, causing them to face significant ‘barriers to being’, including their right to life and protection from discrimination (Franklin et al. 2018b). These can have a negative effect on a family or an individual’s sense of value (Thomas 2004) and even prevent parents and families from seeking support early (Tekola et al. 2020).

## Aims

This study was embedded within a multi-country observational cohort study (Childhood Acute Illness and Nutrition, CHAIN Network) to identify modifiable risk factors for mortality among hospitalised children with acute malnutrition in Africa and Asia (Diallo et al., 2021). Neurodevelopmental assessments for follow up of children who were admitted with severe or moderate acute malnutrition were conducted in three of the sites in LMICs (Pakistan, Malawi and Uganda). All three countries have limited infrastructure for supporting identification and support of children with disabilities. Our objectives were, therefore, to understand the experiences and perceived pathways as well as the barriers and facilitators to identifying children from the; a) perspective of professionals who work with these children in schools, communities and hospitals and b) perspectives of parents of children with developmental disabilities in the early years.

## Methods

### Study design

We used a ethnographic approach to data collection through the use of in-depth interviews (IDIs) and focus group discussions (FGDs) to gain a detailed understanding of experiences, beliefs and opinions of parents, teachers and health care providers (Al-Busaidi 2008, Patton 1990). IDIs enabled us to obtain detailed and often sensitive information from family members and professionals on a one-to-one basis, allowing for privacy (Morris 2015). Whereas FGDs facilitated interaction between participants from similar backgrounds who had similar or varied opinions (Bourgeault, Dingwall, and Vries 2010). Using these different approaches enabled us to triangulate data therefore strengthening the validity of our findings (Patton 1999). Throughout our study, we used the consolidated criteria for reporting qualitative research guidelines to ensure consistent reporting of study aims, objectives, methods and results (Tong, Sainsbury, and Craig 2007).

### Participants and sampling

At each site, purposive sampling was used to identify hospital and community contacts with experience of looking after children with disabilities (parents or professionals) (Ritchie and Lewis 2003). IDIs and FGDs took place at hospitals in the three countries: Queen Elizabeth Central Hospital (Malawi), Mulago Teaching and National Referral Hospital (Uganda) and the Aga Khan University Hospital (Pakistan). We selected participants who had the required characteristics to provide us with the information for which we were searching. These included professionals working with children with disabilities, or parents who had a young child with a disability. Each site identified a slightly different group of participants although they used a similar sampling framework. This is highlighted in the results.

The study population included the following: 1) 20 caregivers of children with disabilities (14 from Malawi and 6 from Uganda); 2) 27 professionals from (a) health; including doctors, physiotherapists, nurses, occupational therapists and clinical health workers, (13 from Malawi, 10 from Uganda and 4 from Pakistan) (b) community health workers (5 from Malawi) and (c) 3) three head teachers from Pakistan, one teacher for children with physical disabilities and two teachers for deaf children in special schools in Uganda and one specialist teacher from Malawi. Separate FGDs were conducted for parents and medical professionals and comprised between 4–8 participants and these varied depending on the options within that country.

### Data collection

Data was collected from January to November 2019 using separate IDI and FGD topic guides for parents/carers, teachers and health care professionals, co-developed by the collaborative research team. We focused on; the perceptions of disability, experiences of identifying children with disabilities and accessing care and support. Topic guides were piloted and refined, incorporating contextual nuances and then translated into Chichewa (Malawi), Luganda (Uganda) and Urdu (Pakistan).

In **Malawi,** FGDs and IDIs were conducted amongst mothers who had formed parent support groups through Malawi Council for the Handicapped and Parents of Disabled Children in Malawi in Blantyre (urban) and Ntcheu (rural) regions of Malawi. Two FGDs with professionals included health care providers with diverse skills for assessing and caring for children with disabilities. All IDIs and FGDs were conducted in Chichewa (parents) or English (health professionals) by a trained female research assistant (RA), who works in QECH as a rehabilitation technician, but also supports the mobilization of caregiver support group meetings in the community.

In **Uganda**, parents/carers were identified for interview through institutions that provide health, rehabilitation and educational services to children with disabilities at Mulago University and Referral Hospital (MURH) in Kampala. Health professionals included occupational therapists and a nurse from the rehabilitation service at MURH. Teachers from the Kampala School for Children with Physical Disabilities and a local non-governmental organization (GEM Foundation), were also invited to participate in the two FGDs. Eight IDIs were conducted in English with representatives from the Ministry of Health, the Ministry of Gender and Social Welfare as well as health care providers working in supporting the health and welfare of children with disability. FGDs were also conducted in English amongst professionals (including nursing officers, occupational and speech therapists) at Mulago hospital’s education and rehabilitation services. Buganda language was used for FGDs with caregivers in the rehabilitation centre and teachers at the School for children with Physical Disabilities. The IDIs and FGDs were carried out by two trained RAs (male and female), with seven years of qualitative research experience between them. One of them also had expertise in child development.

In **Pakistan**, medical professionals were identified for both IDIs and FGDs through colleagues at the Aga Khan University hospital and included rehabilitation staff and doctors. Head teachers of three private schools as well as parents, primarily from Karachi and surrounding urban and rural areas within Sindh, were identified through Paediatric Neurology and rehabilitation clinics. IDIs were conducted in the hospital and in three with headteachers in mainstream schools in Karachi. These were also undertaken by three trained RAs (one a social scientist), all with experience of conducting clinical research in childhood neurodisability.

All IDIs and FGDs were audio recorded. Those in English were transcribed verbatim whilst those in the local languages were simultaneously translated and transcribed into English by the trained RAs at their respective sites. Each RA reviewed the transcripts for accuracy and discussed these with the lead investigators at each of the sites to ensure the validity of the transcripts (Kvale 1996).

The following abbreviations are used to denote the method used: IDI or FGD; the country: Malawi- Mw, Pakistan -PK, Uganda - UG, and the role of the respondent: parent - P, health worker - HW, head teacher - HT, specialist teacher – SPT.

### Data analysis

Data analysis was conducted alongside data collection, allowing for deeper exploration of key emerging themes. (Braun and Clarke 2006). Transcripts were checked for quality and analysed using NVivo 12 software (QSR). A coding frame was created by two members of the research team in the UK (PL, HN) which eventually agreed on eleven themes. PL and HN coded the transcripts and compared codes electronically and then through a consensus meeting prior to final codes being decided upon. Codes were collapsed into a coding framework with main themes and sub-themes.

### Ethical approval

The study was approved by the Research and Ethics Committee at the Liverpool School of Tropical Medicine, UK (18-001), College of Medicine, University of Malawi (P.06/18/2424) Makerere University, Uganda (2018-147) and the Aga Khan University, Pakistan (2018-0271-377). All potential participants received written and verbal information in English or Chichewa (Malawi), Luganda (Uganda) and Urdu (Pakistan) about the purpose and procedure of the study. A written consent form was completed and verbal audio consent before commencing data collection.

## Findings

Our findings are based on the personal views and experiences of individuals (professionals and parents) at each site.

We have separated the themes into major categories; those, which relate to the ‘demand’ for early identification and services by the community and those, which relate to the ‘supply’ of services and support from health institutions and professionals. It is clear that the interplay between these two creates a vicious circle, which prevents any forward movement for change. Limited demand from parents means there is a lack of perceived need for services and without effective services, support and processes for early identification, parents are not compelled to demand more services.

The three themes highlighted by parents and professionals as issues preventing demand relate to (a) Community perceptions of childhood disability (stigma), (b) local beliefs or perceptions regarding causes of disabilities and (c) limited information (both for professionals and parents). The three major themes which we identified as relating to lack of ‘supply’ side included; (a) the perceived inability of services to respond to that need; (b) lack of perceived value and prioritisation of children with disabilities within systems; and (c) lack of consistency and training in tools for identification and referral structures and pathways. We outline these in more detail in the next sections.

### A: Community understanding and ‘demand’ for early identification and support

#### Stigmatisation and feelings of blame/shame

Our analysis identified many personal experiences of stigmatisation reported by mothers of children with disabilities, many of whom described a sense of being victimised, isolated and helpless in all three sites.

In Malawi, parents who had children with disabilities described how they are often ostracised by the community. They described how they felt a need to protect their children by keeping them away from others in the community. ‘*Thinking of a child in a typical village in Malawi, I believe most of them are left in their homes probably locked up inside because their parents are ashamed and don’t want people to know about it, so these children don’t have access to friends, don’t know some of their family members, they have problems with mobility they don’t go to school or church* (IDI HW Mw).

These issues of stigma; including shame and having a sense of personal wrongdoing can create barriers to parents seeking help; ‘…*because of lack of knowledge in the communities and other associated beliefs, these children and their families are isolated’* (IDI HW Mw).

Feelings of blame assignment were highlighted in Uganda, ‘*…for bringing a bad omen (disability) to the family’* (IDI HW UG), thus, preventing parents from feeling empowered to reach out for help. Furthermore, many health professionals in Malawi, described how stigma may lead to negative consequences for mother and child including; family rejection, abandonment by the father (resulting in lack of support and money) and having to ask other members of the family (commonly grandparents) to adopt the child*; ‘I have seen that most of these parents are single mothers, their husbands have divorced them when they gave birth to a child with a disability…so for a mother to raise such a child and has no business nor work to depend on,… it’s difficult to take the child to the hospital or other service providers’* (IDI HW Mw).

In Pakistan, further references to stigmatisation of children with disabilities were highlighted, particularly around the presence of ‘bad omens’. Hospital doctors described how families keep children with developmental disabilities indoors, refusing to let people (and other local children) visit their homes. This stigma affects all levels of society’s socio-economic groups ‘*…if a child suffers from a fever, he will be taken to the doctor, but if he has a speech delay, parents will not seek for help. Even I have observed that elite class (wealthy) people don’t seek help either’* (IDI HW PK). This sense of shame was also highlighted by one headteacher in Pakistan who felt that some parents were reluctant to seek help because of the stigma associated with having neurodisability or mental health condition, often viewed as being inter-related; ‘*…because right now we think that neurological issues are mental, they don’t have causes, it is not a disease’* (IDI SPT PK). This view may further impede referrals to services, where parents are much more reluctant to care for a child who has a ‘mental health disorder’ compared with a child, with what they might see as, a more organic medical condition (e.g. a sensory impairment).

#### Local beliefs on causes and ‘demand’ for traditional treatment for disabilities

Professionals also described cultural perceptions regarding the aetiology of neurodevelopmental disorders in communities, resulting in parents looking for support from traditional healers before accessing, often, unfamiliar, distant and under-resourced health services. They also highlighted the difficulties they encountered, as health workers, when having to explain the reasons behind a child’s disability. Some of the professionals admit to having little knowledge or understanding of childhood disability which could reinforce a lack of trust and perceived benefit of treatments and care. A special schoolteacher in Uganda enunciated how parents believe that witchcraft ‘*…can work ‘more’ than modern science’.* Further, she described how the lack of information or proof that medical systems could help has led to families choosing to use traditional services; ‘…*there is no proof that it needs medical intervention so that is why they have gone to medicine men and women to seek support so that their children can get well’* (FGD SPT UG). This same teacher reported that some children with disabilities in his community wore a chain of herbal medicine around their necks in order to ‘*fight off the disability or get cured’.* He described how his role was to support parents in accepting their child but also in understanding that the child has not been affected by witchcraft: ‘*so they come to believe that they are not related to witchcraft…but it is a process, you give them time and help them to discover the good in their children…and like of course, God accepts us all’*. In Malawi, a health worker explained that ‘*sometimes parents will be given medicine from the hospital…but they may still continue to practise a traditional cure such as bathe a child in herbs provided by the local traditional doctor’* (FGD HW Mw).

Furthermore, language used by the community can add to parents’ despondence about their children’s future. One mother from Uganda, described how she believed she had produced a child known as ‘*omugogo’* (a child who is disabled and can hardly do anything for themselves but just lie still) or ‘*eyaabwe’* (a state of convulsion, common in children with febrile illnesses). This community’s reported lack of trust in the effectiveness of available medical-model treatment options in either alleviating symptoms or improving functionality at home, is common across the country. She added ‘*…they don’t believe that some disabilities can change with medical intervention so they wait to see an example of a somebody who has improved in the first place…it’s really hard for the community to understand that some can get better’* (FGD HW UG).

Health professionals and parents in Uganda and Malawi described instances of mixing allopathic (conventional) medicine with traditional approaches, which may have implications for the effectiveness or risk of either intervention strategy. For example, one mother in Uganda describes her own locally sought treatment for seizures: ‘*I began giving him bitter things (“omululuza” – a treatment for malaria) … ever since then I have been giving him bitter things (for epilepsy)*’. She then talked about giving her child fewer drugs from the clinic: ‘*I told you that I relaxed giving him drugs……and I decided to return on the review date to see what the health care providers have to say’* (FGD PT UG).

#### Community knowledge or awareness of ‘typical’ development and nature of disability

In Uganda, some parents had some understanding of typical child development trajectories based on their experience of caring for their children but also by observing other children in the community. This helped them to identify delayed or atypical development in their children. One mother commented that ‘*…if the child has not sat in the stage where every normal child is meant to be sitting or not standing…that is how I understand’* (FGD UG). Another mother talked more generally about children’s expected development in relation to the number of months and years; ‘*I think that a child has a disability when he fails to develop some sense [see] at a particular stage or fails to do any other thing like standing…. you realise the child has the spinal cord but cannot sit’.* (FGD PT UG).

Overwhelmingly, both professionals in health and education in Malawi and Uganda described the need for more community sensitisation and awareness about childhood disability, as a first step to identifying children with disabilities. In Malawi, a health worker who had received some training on how to recognise childhood disability, commented that; ‘we *have knowledge that these children can benefit from other medical service’* (IDI HW Mw). Teachers and community health workers in both Malawi and Uganda feel that they need to spend considerable amounts of time talking to parents about disability because ‘*…people in their communities discriminate against them and talk bad about them because they have children with disabilities’* (IDI HW Mw).

Both professionals and parents described the important role that health and education professionals, as well as spiritual leaders played in helping parents come to terms with having a child with a disability and being able to refute what they have been told by their community. A health worker in Malawi said they sometimes call on spiritual leaders ‘*to support the parents since some have questions like ‘why me God, why did you give me this child, what wrong did I do?’* (IDI HW Mw) A parent in Uganda said that: ‘*before, whenever I could talk about my child, I always felt like crying all the time… I had tears in my eyes but they (health workers) counselled us and some of them, who have children like ours, brought them and explained to us where they have come from and what they have gone through with their own children.* (FGD IDI UG). Another parent added that: ‘*they (health workers) told us not to hide away children with disabilities from other people but to put them out and make them be loved by other people’* (FGD PT UG).

There was some agreement about how mother groups were seen as good conduits for medical professionals to discuss childhood disability and encourage mothers to share their own concerns and receive help from a health worker or provider. A primary care health worker in Malawi said he preferred working with mother groups ‘*because they are encouraging and supporting each other in terms of care for children with neuro-disabilities’* (FGD HW Mw).

### B. The ‘supply’ of services and support from health institutions and professionals

We now present some of the findings that emerged from the FGDs and IDIs with education and health professionals working in main cities in Blantyre (Malawi), Uganda (Kampala) and Pakistan (Karachi).

#### Knowledge and training of causes of disabilities and their identification among professionals in health and education

##### Professional knowledge

Health workers in all three country sites demonstrated some knowledge about the definitions and causes of neurodevelopmental disabilities in children with many expressing statements such as; ‘*…it is a condition that affected the brain and caused problems with sitting, walking, hearing and seeing’* (IDI HW PK) or it is*’…a condition when a child isn’t achieving his/her developmental milestones and cognitive ability according to his/her chronological age’* (FGD HW MW). They were also able to give examples of how a child could develop a disability as a result of complications at birth, an imbalanced diet during pregnancy, an underlying genetic condition, an accident or trauma during birth that caused an injury or from maternal measles and many more.

Hospital nurses and midwives were described as often being able draw on their experience when assessing whether a child will develop a disability, for example, if a child does not cry at birth. A health worker in Uganda said, ‘*you need to have a wide knowledge about each and every neuro-disability to come up with the right one’.* (HW IDI UG). A nurse in Malawi felt it was important to ‘*alert the mother of abnormal development like irrespective of whether the problem is small or not’* (HW FGD Mw).

#### Use of assessment tools to support and guide identification

Our interviews in Uganda highlighted a general lack of agreement on what tools might support identifying children with disabilities in hospital or community settings. Hospital rehabilitation workers described designing their own forms to assess children; ‘*…to suit the client group you cannot subject the same tool to children and adults so you sit and see what can work for you, which areas do you really need to assess’* (FGD, HW, UG).

There was little reference to standardised tests used, apart from a few doctors who mentioned using detailed developmental assessment tools, such as, Bayley Scales of Infant Development (in Uganda) (Bayley 2006) and the Denver II Developmental Screening Test (in Pakistan) (Frankenburg et al. 1992). One rehabilitation technician referred to the Malawi Development Assessment Tool (MDAT) (Gladstone et al. 2010), but none of the other medical professionals interviewed named any specific assessment tool they used when assessing a child. There was general acknowledgement between community workers in all three sites that they did not have access, training or guidance on tools, which might support them in reliably screening for or identifying young children with disabilities.

Special school teachers in Uganda described using self-made assessment forms, which record areas of functioning and activities such as self-grooming, toileting, requesting for help etc. These forms would be used to help develop a suitable learning and care curriculum for the children when they entered school. In Malawi, health workers described using forms to collect information about the child’s development history, alongside maternal and child health information. Some health professionals in Malawi described how assessment tools can be helpful even for older children in providing some reference standard of ability to use as a comparison ‘*…some parents think that because their child is older but, it doesn’t have to be compared with any other child so this has helped the parents to (know) where exactly their child is.’* (FGD HW Mw)

#### Identification through education settings

In Uganda, teachers described themselves as key persons in creating awareness in the community and becoming a first ‘*port of call’* for parents who are concerned about their children’s behaviour. They might then support parents by conducting ‘baseline assessments’, which may lead to placement at a special school: ‘*as a teacher, you may identify the child with a disability according to the behaviours of a child…but we do little in identifying children from the community’ (*FGD SPT UG). The teachers added that parents of children with disabilities were supporting other parents to take their children to the special schools to receive the type of counselling advice they are unable to receive in their own communities: ‘*so some parents don’t even know what the challenges are or where those children are really required to be placed, so we so we do a bit of identification of the disabilities ourselves’* (FGD SPT UG).

In Pakistan, a headteacher of a private school suggested organising awareness programmes and seminars to increase parental knowledge and awareness of ‘neurological issues’ such as being able to manage children’s behaviour, ‘mental health’ issues. She also suggested having counsellors trained in neurodisability based at schools who can talk to both teachers and parents about the causes and treatments of different conditions.

#### Provision of services for children with disabilities

### Lack of referral pathways and support structures for families

How children are referred to medical or rehabilitation services in the three country sites was variable. In Malawi, no clear system seemed to be in place for referring children who were identified in the community. Health workers describe the need to strengthen systems including outreach clinics, which would reduce the burden on travel. In our study, health workers described not feeling sufficiently qualified or resourced to provide advice or treatment for children with specific medical needs: ‘*when a family has a child with a disability, they feel like it’s a burden if they come to you for help and you refer them to other providers, it is like adding another burden on them’* (FGD HW MW). Health workers described the lack of up-to-date contact details of service providers or organisations that deal with disability: ‘*I think that could be quite helpful, so that coming up with contact persons all over starting from the communities to the districts and to the level of central hospitals’.* (IDI, HW, Mw).

In Uganda, health workers describe the responsibility being placed on parents to initiate the process and that this depended on their’ levels of ‘motivation’ and ‘passion’ to seek help for their child. It was also noted that where the outcome of a management strategy showed positive change, made a difference to families in terms of maintaining visits for therapy; ‘*so if they see good results it will also motivate them to come back…it keeps them motivated, they yearn for more’* (FGD HW UG).

The structural issues relating to poor referral pathways and guidelines were also highlighted in Pakistan where doctors stated that ‘*referral pathways are very weak’* and children do not always receive follow up appointments from specialist services at central facilities because of the lack of good follow-up systems. A health worker felt that parents are reluctant to go to a different clinic for referral. Children with disabilities ‘*often did not turn up and is time consuming for health care providers’* (HW IDI PK). Even when children do arrive for an appointment, they seldom have the referral reference number, which allows the referral doctor to access information about the child’s condition on the system.

The issue of transport costs for parents to take their children for therapy at central facilities is a challenge for families in Malawi. Parents talked about having to travel long distances and not having sufficient funds to pay for transport to the clinic or hospital: ‘*may fail to go to the hospital and we may not have the money for transport’.* Another parent said that they ‘*sometimes rely on some relations’* or ‘*there should be a place where they can spend the night and get proper meals* (IDI HW Mw).

## Discussion

In this study, we aimed to understand the views and experiences of health professionals, educators and parents in Pakistan, Malawi and Uganda in relation to barriers and facilitators for early identification and support of pre-school aged children with disabilities.

We identified a wide range of experiences of parents, teachers, health and (para)-professionals working in communities across all the three countries. Some themes were apparent to all three countries, however in some instances, a specific theme has more relevance in one or two countries and in these cases, we have highlighted this in the text. Our analysis identified themes which are linked to a host of barriers and facilitators, not just at the level of the community, but also at a systems level (both health and education). Testimonies show that there are communities seeking locally based solutions to local problems with pockets of good practice. There are good examples where parents are motivated to seek ways out of their situation (e.g. seeking advice from a local school) but, the supply side at community level, is under-resourced and unable to meet their needs.

Children in their earliest years of life are changing rapidly and parents and professionals need to have a basic knowledge of child development (National Academies of Sciences et al., 2016). They need to understand specific signs and symptoms of neurodevelopmental delay (National Academies of Sciences et al., 2016, Hagan, Shaw, and Duncan 2017), which is often more culturally driven than knowledge based (Ayob, Christopher, and Naidoo 2021, Garg et al. 2017). Furthermore, when parents do not know what the options are for supporting their child with a disability, this can leave them feeling disempowered and unable to move forward (Hohlfeld, Harty, and Engel 2018).

**Figure 1.**
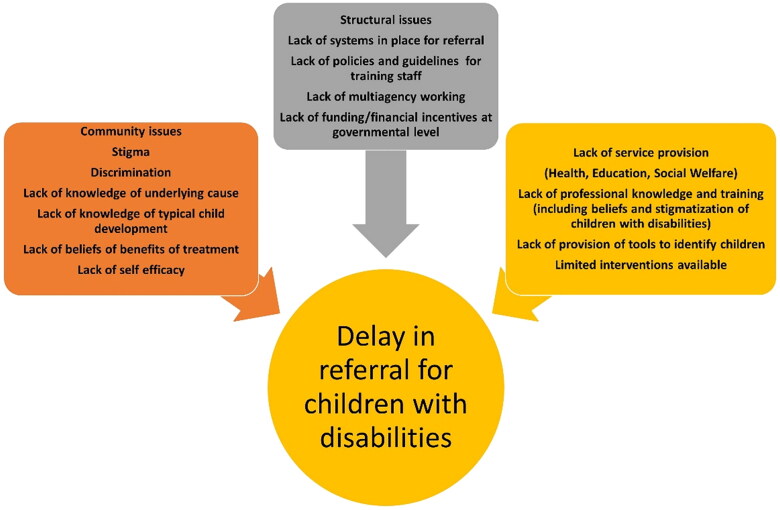
Addressing community, structural and service provision issues for disability at community level placing the child at the centre.

**Figure 2.**
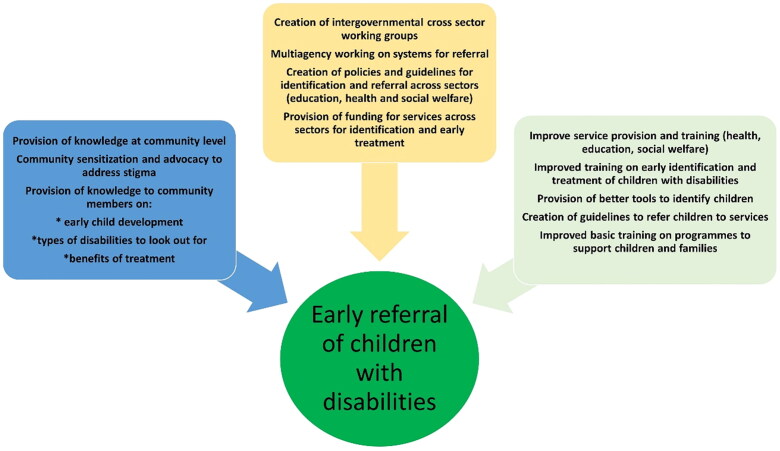
Addressing the demand in knowledge and service provision at community level – placing the child at the centre.

On reflection, we commenced this piece of work aiming to identify the issues with training and resources for professionals who may be in a position to identify children with disabilities. We have clearly identified that there is a complex interplay of many factors which affect early identification of children with disabilities – many of which are linked to the social constructs of disability. With a broader understanding of the child within society, it is clear that, by only addressing issues relating to training professionals in early identification of children with disabilities through tools to support and guide them, we will not deal with the structural and social issues which affect ‘demand’ for services. We demonstrate this in Figures 1 & 2, where we keep the child in the centre of our model. In order to identify the factors in our study, as influencing the identification and referral of children with disabilities. It is clear there are many factors that lie within communities who may resist having their children identified due to stigma, feelings of blame, lack of knowledge and perceptions about treatment options and unclear referral pathways and the lack of multisector working specifically in this area. Furthermore, the lack of specific tools and training for identifying children is certainly an issue, but, without good affordable family intervention programmes, there may be less incentive to address this issue.

Our study has demonstrated that the high levels of stigma relating to children with disabilities across the three country contexts remains a significant barrier to identification and care. In all country sites, there was a strong sentiment that stigma towards disability is still widely prevalent in communities and that lack of awareness and knowledge regarding the causes of different conditions may impede the early identification and referral of children in their early years. Moreover, it can also prevent or delay treatment and rehabilitation interventions at primary and tertiary level. Limited understanding of the lives of families with disabilities in the local context and lack of attention given to the specific needs of children with disabilities means that identification and assessment to better support community awareness and intervention remain elusive (Franklin et al. 2018a).

Views of disability, often describing persons with disabilities as being responsible for or deserving of their disability and provision of causative explanations such as an perceived wrong-doing (Grischow et al. 2019), create resistance to identification and hence a barrier to these families being offered support. In many cases, transgressions are assumed to be of the mother or from the mother’s side of the family being devastating for both the child and the mother and preventing them from feeling they should access community health services. In order to make any headway in improving early identification and support of children with disabilities, we must address and tackle these issues as part of any programme. Any programme, however, needs to be flexible in the understanding and interpretation of stigmatisation, as this will vary from one country context to another and from one area of the same country to another. Understanding the belief systems at work in a community will however be critical to supporting the community in providing access and moving towards greater chances of children with disabilities being identified and referred into supportive health and care management systems. This may be done through a number of means including parent groups which address the conflict between different conceptual belief systems, support parents in understanding their child’s condition and give opportunities to connect with non-governmental support and to rights-based organisations.

### Strengthening parental trust in getting health care

Barriers to parental perceptions around the effectiveness of available care for children with disabilities was highlighted by health and education professionals as being a widespread issue that prevented parents from following the advice of medical doctors and prescribed treatment. Furthermore, structural issues with allocation of resources and finances are also relevant. If parents are unable to access clinics because of long distances and cost of transport, they may be more likely to go to a local traditional healer who can prescribe them with a treatment which they have heard about from others in the community. The lack of access to or clear information about the goals or effectiveness of a particular treatment for children with disabilities is likely to lead to high levels of non-attendance at health clinics.

Finding ways of increasing parental trust as to the value of identifying and supporting children and families earlier may change this. Some of this might be supported through building stronger awareness through training community health workers on interventions to support children with disabilities. One way is to invite model champions with disability to share their experiences of using a specific intervention or a care management plan. A lack of knowledge by parents in seeing differences between children’s rates of development did not seem to be an issue, moreover, this knowledge seems to be widely known across the community but not widely acted upon.

### Better coordination between referral services

We have not identified many examples of good practice in identification and referral services within our ethnographic study. Practices emerge from the transcripts in this limited cohort as being rather haphazard and left to those parents who are determined to seek the right treatment for their children. No professional or parent referenced any screening practices that were taking place in the three countries. Furthermore, it is evident from some viewpoints, that even when children were being referred to services, the correct information was not always following the child to the hospital. This lack of coordination between community and health services may lead to further entrenching of pessimistic views of referral procedures, generating a cycle of mistrust and parental take-up of vital health services. (Koerting et al. 2013). The most vulnerable families are often the hardest to reach or remain invisible to essential services. It is important that these families living in rural areas can benefit and receive help from local service providers (Lynch, Lund, and Massah 2014).

Health workers see the need to strengthen outreach clinics in the community, however, it is likely that low levels of staffing do not lead to effective change in the low levels of trust in communities. Staffing clinics with rehabilitation officers or health workers, who, have received little or no training in how to identify and support children with disabilities, may not be able to shift a continued community mistrust. The results from our interviews and focus groups suggest that many health workers do not feel sufficiently qualified to advise parents on what treatment their children may need. This may be due to the type of training programme they have received and the amount of content on identifying early childhood disability. Building on tools that may link guidance and support to identification of children with disabilities is evidently needed. Some new incentives are underway and a revision of the WHO mhGAP – ‘Caregivers Skills Training Programme for Families of Children with Developmental Disorders or Delays’ (Salomone et al. 2019) may help provide some guidance here. Furthermore, WHO ‘Care for Child Development’ does advocate use of its materials for any child without needing to specifically have a clear level of development of the child. This perspective is important if we want to gain trust from communities through demonstrating supportive interventions that can be carried out at basic community level. In addition, we need to identify the key characteristics of an effective integrated working system which includes having a shared vision, clear understanding of needs, identification of gaps and a sharp focus on improving outcomes for children with disabilities. We also need to develop strategies that will ensure buy-in from families and communities on what they deem to be important for their children’s development and well-being.

### Potential solutions. Who should identify children, where and when?

Clearly, stepwise, incremental approaches provided at all levels including families, community health workers, as well as higher levels of services are likely to work best in low resource settings. This stepwise approach might include. Thinking through good strategies of putting these into place is important. These may include the use of a short set of questions at immunisation clinics or through child health cards or parent health records –techniques which are already utilised in many settings, although demonstration of efficacy is limited therefore piloting would be recommended to demonstrate good uptake and follow-up.

In a number of settings, it is clearly important to consider whether the best conduit for early identification may be through educational and early years’ settings rather than through health clinics. Probably both, working hand in hand, are important. Educational establishments may have much more of a bi-directional approach reducing the chances of referred children not receiving essential support, but also receiving the input they may need from health and then coming back into supportive education.

These findings will inform the proposed implementation of a global tool to identify neurodevelopmental disabilities among children aged 0 to 3 years in Malawi, Pakistan, Uganda and other similar contexts (https://www.liverpool.ac.uk/translational-medicine/research/indigo-study/contact/).

## Study limitations

In this study we aimed to understand the experiences and perceived pathways, barriers and facilitators to identifying children from the; a) perspective of professionals who work with these children in schools, communities and hospitals and b) parents of children with developmental disabilities in the early years. The analysis drew upon the societal, cultural and historical contexts as experienced and understood by the participants. The authors are mindful of the caution that has been raised against research in LMICs that is dominated by descriptions of negative attitudes while failing to acknowledge the continuum along which these attitudes and perceptions exist. It is possible that contemporary African countries such as Malawi and Uganda now view disability through a more pluralistic lens that sees disability as a synthesis of traditional and biomedical explanations. This is particularly difficult when trying to find the correct interpretation for specific words and expressions related to traditional beliefs and superstitions in Malawi and Uganda. We acknowledge that the findings from the study represent a segment of reality in the three country sites, and there are no claims regarding capture of the cultural context of childhood disability in its entirety. In an attempt to recruit similar groups of participants in each site, we tried to obtain as much of a balanced snapshot of what is happening in the three countries. We were unable to recruit as many participants in Pakistan as in the other sites, which means that we only have a glimpse of how childhood disability is understood within an urban context and at a privately run hospital and schools.

We would need to carry out a more in-depth study in Malawi and Uganda to allow for a deeper understanding of how Africa’s colonial past has affected current cultural norms and practices related to disability. Another limitation is that we did not speak to policymakers to understand how resources are allocated to the identification and management of children with neurodevelopmental disabilities.

## Conclusion

This study has highlighted the need to strengthen pathways for early identification and intervention of children with neurodevelopmental disabilities in these three settings in LMICs (Scherzer et al. 2012). This entails empowering families and communities to identify problems and to seek help. It also requires the strengthening of community health workers’ training and skills in child development screening. The collection of data to monitor referral patterns and identify gaps as well as build linkages between health system levels and across sectors including education and social welfare services (Yousafzai, Lynch, and Gladstone 2014) is required. There is also an urgent need to review policies and incentives to improve supply structures through better systems referral, multiagency approaches (social welfare, education and health) and increased funding in these LMICs. Finally, effective monitoring of systems to ensure good fidelity of a piloted strategy is required.
